# Review of T cell proliferation regulatory factors in treatment and prognostic prediction for solid tumors

**DOI:** 10.1016/j.heliyon.2023.e21329

**Published:** 2023-10-29

**Authors:** Jiayu Li, Shuhan Ma, Hongdi Pei, Jici Jiang, Quan Zou, Zhibin Lv

**Affiliations:** a Student Innovation Competition Team, College of Biomedical Engineering, Sichuan University, Chengdu 610065, China; b College of Life Science, Sichuan University, Chengdu 610065, China; c Institute of Fundamental and Frontier Sciences, University of Electronic Science and Technology of China, Chengdu 610054, China; d Yangtze Delta Region Institute (Quzhou), University of Electronic Science and Technology of China, Quzhou 324000, China

**Keywords:** T cell proliferation regulators, Solid tumor, CAR-T, Immunocyte therapy, Cancer prognosis prediction, T cell proliferation and activation

## Abstract

T cell proliferation regulators (Tcprs), which are positive regulators that promote T cell function, have made great contributions to the development of therapies to improve T cell function. CAR (chimeric antigen receptor) -T cell therapy, a type of adoptive cell transfer therapy that targets tumor cells and enhances immune lethality, has led to significant progress in the treatment of hematologic tumors. However, the applications of CAR-T in solid tumor treatment remain limited. Therefore, in this review, we focus on the development of Tcprs for solid tumor therapy and prognostic prediction. We summarize potential strategies for targeting different Tcprs to enhance T cell proliferation and activation and inhibition of cancer progression, thereby improving the antitumor activity and persistence of CAR-T. In summary, we propose means of enhancing CAR-T cells by expressing different Tcprs, which may lead to the development of a new generation of cell therapies.

## 1 Introduction

Solid tumors represent a type of clinically diagnosed tumors and include malignant solid tumors and benign solid tumors. Benign solid tumors include leiomyoma [1] and lymphangioma [2], whereas malignant solid tumors include mesothelioma [3], colon cancer [4], and ovarian cancer [5,6]. In contrast to non-solid tumors such as hematologic tumors, solid tumor masses can be detected by X-ray, computed tomography scan, ultrasound, or palpation. In recent years, tumor immunotherapy for the treatment of solid tumors has become a research trend.

The first immunotherapy drug, “Coley's Toxins,” invented by the surgeon Coley, was introduced at the end of the 19th century. It is composed of inactivated streptococcus and salmonella, and tumor regression can be observed after its intratumoral injection. Since then, tumor immunotherapy has flourished. Tumor immunotherapy aims to treat cancer by artificially intervening in the body's immune system to enhance or decrease the immune response [7]. It is usually divided into two categories: active and passive immunotherapy [8]. The former type, which includes tumor vaccines, actively stimulates the immune system to enhance the immune response and eliminate cancer cells, whereas the latter type causes the body to passively accept antibodies, cytokines and includes adoptive cell therapy, oncolytic viruses, and monoclonal antibodies.

Adoptive cell transfer therapy is a treatment in which autologous or allogeneic immune effector cells, activated and amplified in vitro, are infused into the patient's body. It usually consists of TIL (tumor infiltrating lymphocytes), TCR-T (T cell receptor-gene engineered T cell), or CAR-T (chimeric antigen receptor T cell) therapy regimens. CAR-T therapy has shown great potential in the treatment of malignant hematologic tumors, and TIL and TCR-T therapies have shown some effectiveness in a small number of solid tumors. However, their benefit to patients remains limited for most solid tumors, mainly owing to factors such as antigen heterogeneity and immune escape, poor infiltration ability of immune cells, immunosuppression of the tumor microenvironment (TME) and metabolic microenvironment obstruction, and T cell exhaustion [[9], [10], [11]]. CRISPR-based loss-of-function screens are currently limited to negative regulators of T cell function [[12], [13], [14], [15]], and positive regulators of T cell function remain undetermined.

On March 16, 2022, Neville E. Sanjana's group and Mateusz Legut collaborated to search for positive regulatory factors that promote T cell function using genome-wide large-scale screening. These factors were selected by CD3/CD28 stimulation, and therefore have an intrinsic role in T cell proliferation. They identified 35 top-ranked genes that promoted proliferation and activation of human CD4 and CD8 T cells, of which LTBR had the most significant enhancing effect on T cell function [[16], [17], [18]]. CAR-T cells can exert clinical effects by expressing certain genes, for example, cytokines such as interleukin 12 (IL-12) or interleukin 18(IL-18) [19,20]. Thus, enhancing CAR-T cells using these top-ranked T cell proliferation regulators (Tcprs) may lead to the development of a new generation of cell therapies. This article reviews the latest research findings and trends in this field, focusing on the use of Tcprs in solid tumor treatment and prognostic prediction.

## 2 Definition of TCR signature

In a study published in March 2022, Mateusz Legut used large-scale TCR functional screening to show that MAPK3, CD59, transcription factor BATF, and cytokines such as IL12B and IL23A could promote T cell proliferation; the 35 top-ranked ORFs (open reading frames) (Table 1) improved T cell proliferation while promoting cytokine secretion [16]. This was the first genome-wide screen for positive regulators of T cell function and is expected to pave the way for clinical application studies of Tcprs.

**Table 1:** T cell proliferation-associated regulators [21].

T cell proliferation-associated regulators						
IFNL2	IL12B	ATF6B	CLIC1	CDK1	DUPD1	ADA
LTBR	NFYB	AHNAK	RAN	DBI	GPD1	ITM2A
IL1RN	BATF	SLC10A7	CDK2	CYP27A1	GPN3	HOMER1
CXCL12	FOSB	CALML3	MS4A3	AKR1C4	AHCY	MRPL51
LIG3	ZNF830	DCLRE1B	B2M	HLA-A	CD19	NGFR

The researchers also found that overexpression of LTBR and other top-ranked genes could induce extensive transcriptome changes in T cells, accompanied by changes in T cell function [16,22]. This suggests that these genes could be used to improve the CAR-T cell response of healthy T cells or to improve the response of dysfunctional T cells of patients. The results thus provide new ideas for improving response and cure rates in cancer immunotherapy through armoring CAR-T cells by expressing TCR genes to increase their anti-tumor activity, enhance T cell killing, and delay CAR-T cell depletion.

## 3 Tcprs and treatment of solid tumors

T lymphocytes are derived from pluripotent stem cells from the bone marrow, which mature and differentiate in the thymus into two major subpopulations, CD4^+^ T cells and CD8^+^ T cells. Upon activation, they differentiate into helper T cells (Th) and cytotoxic T lymphocytes (CTL), respectively. T cells contribute to the cellular immunity of the body by eliminating cells infected with viruses or bacteria, as well as tumor cells, and also secrete lethal cytokines. They play an important part in anti-tumor immunity by monitoring mutations in the body and resisting infection from outside the body.

T cells cannot function without their activation mechanisms. Three signals are required for the proliferation and full activation of T cells. The first is the antigen-stimulated signal generated by binding of human leukocyte antigen (HLA) on the surface of immune cells, histiocytes and cancer cells to T cell receptors and co-receptors, which is acquired and specific. The second signal is a non-specific costimulatory signal, which is mainly generated by the interaction between costimulatory molecules on the surface of antigen-presenting cells (APCs) and the corresponding receptors of T cells. The third signal is the cytokine secreted by the cell, which stimulates an intracellular signaling cascade after binding to the cytokine receptor, causing the proliferation and differentiation of T cells [23]. All three signals are indispensable. In the absence of a costimulatory signal, the first signal fails to produce specific activation and may even deactivate T cells; this not only increases energy consumption but also has unpredictable negative effects. In the absence of cytokines, T cells can expand but cannot exert a powerful killing effect [24].

Many Tcprs have been shown to affect these mechanisms, a finding that has great significance for T cell proliferation and activation and tumor clearance. CAR-T cell therapy, which artificially and specifically activates CD8^+^ T cells, is currently the most promising technology for tumor immunotherapy. Thus, the addition of Tcprs to CAR-T therapies may drive breakthroughs in the treatment of solid tumors. Below, we elaborate on the relevance of the abovementioned 35 Tcprs to CAR-T, which is known to be a promising therapy for solid tumors.

### 3.1 CAR-T cell therapy is expected to become a new paradigm in the treatment of solid tumors

CAR-T cell therapy is a type of cellular adoptive immunotherapy in which activated T cells are genetically engineered with a CAR to target tumor cells and enhance immune lethality. CAR-T cell therapy has been widely used in the clinical treatment of malignant hematologic tumors and has shown good efficacy [[25], [26], [27]], which highlights its great potential as a tumor treatment. However, CAR-T has shown limited efficacy in the treatment of solid tumors [[28], [29], [30]].

CAR is a fusion protein expressed in T cells. Its greatest advantage is that it can bypass the constraints of the histocompatibility complex (MHC) and bind directly to the surface of cancer cells to induce death. CAR-T cells are mainly composed of three components: an extracellular domain that recognizes tumor antigens (SCFV fragments); a transmembrane domain (CD8); and an intracellular domain that mediates T lymphocyte activation (costimulatory molecules) [31]. In addition, CAR constructs also include immunoreceptor tyrosine-based activation motifs (ITAMs) derived from CD3zeta, which serve as the most important and core signaling element [32]. The mechanism by which CAR-T cells play their part in tumor tissue can be summarized as follows. SCFV fragments bind specifically to antigens on the tumor cell surface to generate the first signal required for T cell activation. The activation signal then reaches the intracellular costimulatory domain through the transmembrane domain and generates a second signal to activate T cells through the activation of costimulatory molecules. Eventually, the T cells participate in the killing of tumor cells [33]. It is thus clear that to improve the effectiveness of CAR-T it is necessary to improve the function of the T cells themselves. Four generations of improvements have been made to solve this problem. The first generation only provides the first signal. The addition of multiple co-stimulatory structural domains in both the second and third generations enhanced and maintained secondary signaling by comparison, but the issue of persistence of T cell anti-tumor activity was not altered. The fourth generation added co-expressed cytokines, in contrast to an enhanced third signal, which not only improved the expansion and persistence of CAR-T cells but also improved their function in the immunosuppression of the TME.

Although there have been many attempts to expand the scope and improve the applications of CAR-T so that it can be more quickly and effectively applied in the clinical treatment of solid tumors, there are still three major obstacles to improving the anti-tumor activity of CAR-T cells. These are described in the following sections.

#### 3.1.1 Target antigen selection

Unlike hematologic tumors, solid tumors have heterogeneous target antigens. Many tumor-associated antigens are co-expressed in normal and tumor tissues, and misdirected antigens can lead to normal cell killing and severe toxicity. Injection of large numbers of CAR-T cells at once could cause a cytokine storm and possible “off-target toxicity” to normal cells [34]. This significantly increases the risks associated with CAR-T therapy. Therefore, finding an antigen that can specifically recognize different tumors is of great significance for CAR-T therapy.

#### 3.1.2 Proliferation and persistence of CAR-T cells

T cell depletion is a dysfunctional state of exhaustion that, unlike aging, is associated with progressive loss of effector function and poor memory T cell response [34]. Depleted T cells have low capacity for proliferation and cytokine secretion and high levels of apoptosis [35]. In many cases, it is not that the input of CAR-T cells is insufficient or that they are not lethal enough, but that the exhaustion of CAR-T cells makes their efficacy very poor. Targeting transcription factors is associated with CAR-T cell persistence. Studies have shown that CAR-T cells lacking NR4A1, NR4A2, and NR4A3 transcription factors have downregulated PD-1 and TIM3 expression and stronger anti-tumor effects [36]. Second-generation CAR-T also promotes T cell proliferation and persistence owing to the addition of costimulatory signal domains from CD28 or 4-1BB [37]. Therefore, maintaining T cell proliferation and persistence is critical for the long-term clinical efficacy of CAR-T.

#### 3.1.3 Tumor microenvironment

The extracellular matrix of cancer contains a broad collection of genetically normal cells, collectively called the TME [38]. Owing to the existence of the TME, it is difficult for CAR-T cells to physically penetrate severely fibrotic tumor tissue, and some tumors inhibit chemokine signaling that helps to mediate T cell infiltration [39]. Chemokines are a major determinant of the degree of cytotoxic T cell infiltration in solid tumors and can recruit immune cells into the TME [40]. Many solid tumors abnormally express chemokines to evade immunity. Dysregulation of chemokines in the TME contributes to tumor proliferation and metastasis [[41], [42], [43]]. The TME preferentially recruits suppressive immune cells, such as regulatory T cells and myeloid-derived suppressor cells, but prevents effector lymphocyte recruitment and induces an anoxic microenvironment [[44], [45], [46]]. Thus, targeting chemokine networks can promote anti-tumor immune responses, such as improved homing of CAR-T cells with greater antitumor activity through co-expression of chemokine receptor CCR2 [47].

### 3.2 Strategies to enhance CAR-T with tcprs

CAR-T therapy is a new “star” in cancer therapy; its potential is evident and is becoming clearer with its widespread use. T cell modification may be a starting point to enhance CAR-T cells by improving their proliferation, activation, and persistence. It may also be possible to design specific targets for different tumor cells to make CAR-T truly a “site-blasting” tumor killing weapon.

The top-ranked gene set of Tcprs has an impact on all three aspects of the barriers to CAR-T cell therapy development. The direction and extent of the effects are related to both the mechanisms by which different genes act and the type of tumor. The following sections focus on three aspects of enhancing the anti-tumor activity of CAR-T cells to explain the possible role of TCR and the mechanism of action.

#### 3.2.1 Enhancing T cell proliferation and activation

According to the literature we found that there are seven Tcprs have a potential role in promoting both proliferation and activation of T cells, of which two promote activation only, one promotes proliferation only, one TCR inhibits T cell proliferation, and two exhibit properties associated with immune cells (Table 2).

**Table 2:** Effects of TCR on T cell function.

Gene	T cell proliferation	T cell activation	Associated with immune cells
CXCL12	+	+	/
LTBR	+	+	/
CDK2	+	+	/
AHNAK	+	+	/
IL1RN	+	+	/
CD19	+	+	/
ADA	+	+	/
RAN	/	+	/
CLIC1	/	+	/
ITM2A	+	/	/
BATF	–	/	/
B2M	/	/	✓
CDK1	/	/	✓

**Fig. 1:**
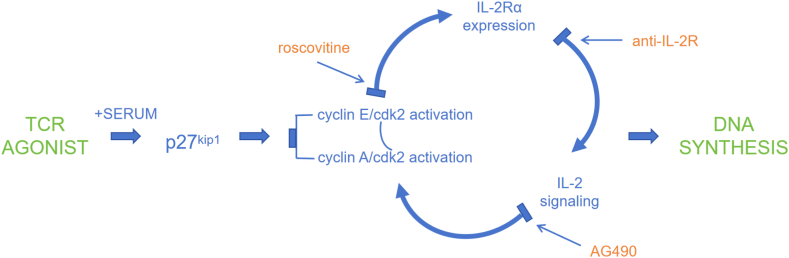
Interdependence of cdk2 activation and IL-2 signaling [50].

T cell proliferation and activation are particularly important for the immune system. Borge et al. showed that CXCL12 is a co-stimulatory factor for CD4^+^ T cell activation and proliferation in patients with chronic lymphocytic leukemia [48]. The increased expression of activation markers CD25, CD69, and CD154 suggests that CXCL12 may play an important part in the effective activation of T cells [49]. Legut et al. overexpressed LTBR in T cells and found that the secretion of cytokine IL-2 was increased and activation-induced apoptosis was reduced to a certain extent, resulting in resistance to partial T cell depletion [16]. T cell proliferation is controlled by the sequential activation of cyclin-dependent kinases (CDKs). As CDK2 activity contributes to lymphokine IL-2 signaling, which itself is generated by IL-27 signaling, Mohapatra et al. proposed a T cell proliferation model in which CDK2 activation, IL-2Rα accumulation, and IL-2R signaling form a feedforward loop [50] (Fig. 1). This suggests that CDK2 may be involved in the regulatory pathway of T cell proliferation. AHNAK, also known as AHNAK1, is a scaffold protein highly expressed by CD4^+^ T cells and is a key component of calcium signaling. Matza et al. found that CD4^+^ T cells in AHNAK1-deficient mice had poor response to T cell antigen receptor stimulation in vitro, with low proliferation and low secretion of the cytokine IL-2 [51]. Other studies have shown that an increase in AHNAK levels in CD4^+^ T cells may promote the production of cytokine IL-6 [52]. Inflammatory response models have shown that T cell activation requires APC-derived IL-1 [53]. Matsuki et al. investigated the IL-1/IL-1Ra system induced by myelin oligodendrocyte glycoprotein and found enhanced production and proliferation of IFN-γ, IL-17, and tumor necrosis factor-α in IL-1Ra^−/−^ T cells [54]. IL1RN, also known as IL1RA, may have the potential to promote T cell proliferation and activation. CD19 encodes a member of the immunoglobulin gene superfamily, and this protein serves as a target for CAR-T cell therapy. Ung et al. developed a CD19CAR structure and constructed a novel co-stimulatory domain [55]. They found that CD19CAR-T cells had significant upregulation of genes associated with T cell activation, proliferation, interferon production, NF-κB signaling, and memory signatures and downregulation of apoptosis and T cell depletion genes compared with CD28 or 4-1BB cells [55]. The CD19CAR, which recognizes CD19, may promote T cell proliferation and activation while enhancing CAR-T cell persistence by downregulating T cell depletion. ADA encodes an enzyme that catalyzes the hydrolysis of adenosine to inosine in the purine catabolic pathway. Soluble immunoregulatory proteins of human plasma include adenosines deaminase 1 (ADA1) and ADA2 [56]. ADA1 deficiency impairs thymocyte development and B-lymphocyte immunoglobulin production, leading to severe combined immune deficiency [57]. ADA2 also induces the differentiation of monocytes into macrophages in T cell co-culture [58]. ADA2 deficiency results in inflammatory conditions as well as changes in the distribution of immune cell subsets and immunoglobulin levels [[59], [60], [61]]. Cortes et al. showed that ADA-catalyzed intracellular and extracellular purine metabolism can play an important part, and that ADA acts as a co-stimulator to promote T cell proliferation and differentiation by interacting with differentiation cluster CD26 [62]. In addition, Casanova et al. showed that ADA-induced pro-inflammatory cytokine secretion could provide a local “suppression-free” environment to promote T cell activation [63]. They found that the addition of ADA not only promoted the secretion of cytokines but also increased the secretion of chemokines including CXCL8, CCL2, CCL3, CCL4, and CCL5 [63]. These chemokines recruit immune cells such as neutrophils, monocytes, dendritic cells, and activated T cells to the antigen-treated site to further develop the immune response [64]. All of the above genes show a non-negligible potential to promote both T cell proliferation and activation, and they may show surprising effects in TCR-enhanced CAR-T.

**Fig. 2:**
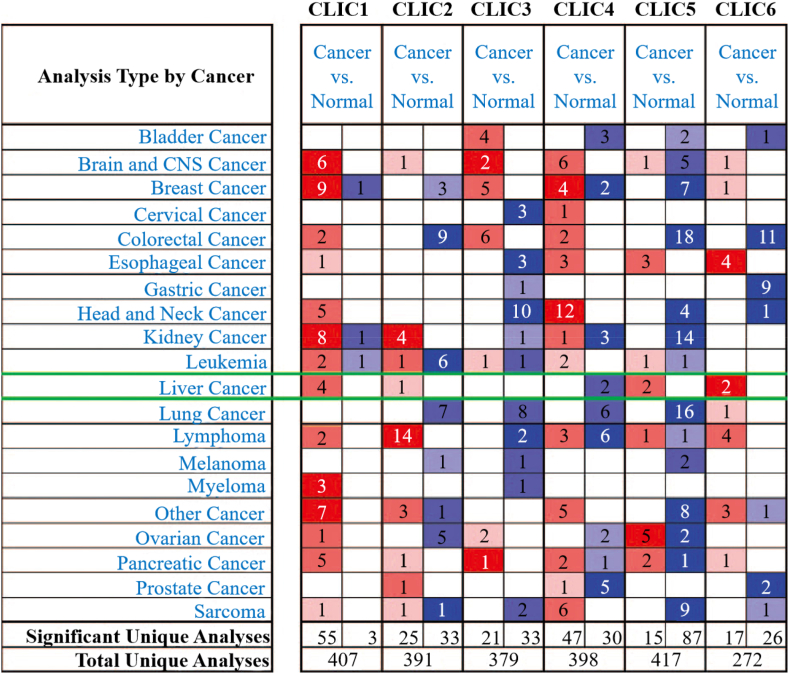
Transcriptional expression of CLICs in 20 different types of cancer (ONCOMINE) [68].

T cell activation requires the combination of T cell antigen receptors and co-stimulatory receptors, but many tumor cells cannot express the corresponding ligands. Nieland et al. found that gene-transfer-mediated RAN/TC4 elevation could introduce a co-stimulatory function of CD8^+^ T cells into tumor cells [65]. Chloride intracellular channel 1 (CLIC1), a gene highly expressed in macrophages, encodes a member of the highly evolved conserved chloride channel protein CLIC family and was first cloned owing to its increased expression in activated macrophages [66]. Macrophages are key cells in innate and adaptive immunity, with a crucial role in defense repair and tissue homeostasis [67]. However, CLIC1 participates in the inflammatory process by regulating macrophage phagosome function (e.g., pH and proteolysis), and its high expression is associated with a variety of immune cells including B cells, CD4^+^ T cells, and macrophages [68] (Fig. 2). Therefore, CLIC1 may promote T cell activation. In conclusion, the above two genes (RAN and CLIC1) are likely to be involved in promoting T cell activation, providing insight into how different Tcprs could be used to improve CAR-T.

ITM2A, a novel marker of T cell development, was identified as an MHC-mediated double-positive thymocyte-positive selection gene [69] that is expressed more highly in the thymus than in the spleen or lymph nodes. Kirchner et al. demonstrated experimentally that overexpression of ITM2A led to an increase in the number of CD4^+^ T cells and CD8^+^ T cells [70]. ITM2A is a type II transmembrane glycoprotein expressed on the cell surface [71]. However, Tai et al. studied the relationship between ITM2A protein and T-cell-specific transcription factor GATA-3 and found that the lack of ITM2A had little effect on the differentiation, activation, or function of T cells in vitro. This may be related to the compensatory effect of ITM2B expression in T cells [69] (ITM2B also belongs to the type II integrated membrane protein family, which is highly expressed in T cells). Therefore, ITM2A, as a TCR, may be related to the promotion of T cell proliferation.

B-cell-activating transcription factor (BATF) can inhibit T cell proliferation. BATF belongs to the activator protein-1 (AP-1) superfamily of basic leucine zipper transcription factor and forms heterodimers with minimal transcriptional activity with Jun to produce complexes with little transcriptional activity when bound to AP-1 target genes [72]. Thornton et al. stimulated transgenic thymus cells expressing BATF under different conditions and observed impaired T cell proliferation under all conditions [73].

**Fig. 3:**
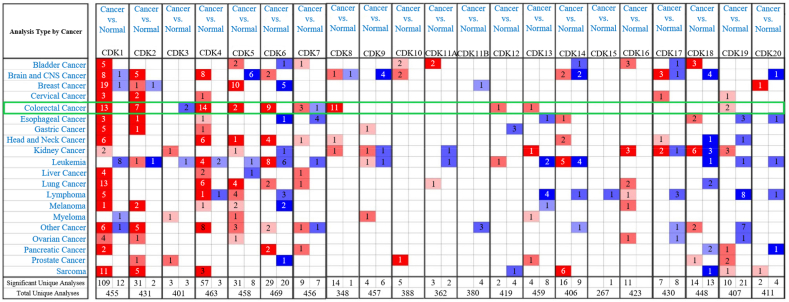
Expression of CDK family members in different cancer types [76].

Some genes have not been shown to promote T cell proliferation or activation but have properties associated with immune cells. The human immune response is associated with cell surface HLA. For example, HLAI molecules initiate the immune response during transplantation by presenting antigens to CD8^+^ T cells. B2M molecules are known to bind to HLAI molecules, and the expression of HLAI molecules on the cell surface must bind to B2M [74]. Meshitsuka et al. found that the expression of HLAI molecules could be inhibited by knockout of B2M, so that the cells could escape the immune response mediated by T cells [75]. B2M gene knock-out in combination with B2M-HLA-G knock-in protected cells from both T cells and NK cells, which shows potential in suppressing allograft rejection. Tumor occurrence and progression are closely related to tumor infiltration of lymphocytes. Expression levels of all CDKs except CDK16 are correlated with infiltration of immune cells such as CD4^+^ T cells, CD8^+^ T cells, B cells, and regulatory T cells, including CDK1 in Tcprs (Fig. 3) [76]. Guan et al. in a study of CDKs, demonstrated that CDK1 was associated with low invasion of regulatory T cells and prognosis in colorectal cancer, indicating that high expression of CDK1 is associated with good prognosis of colorectal cancer patients [76].

The above properties of TcprsTcprs can be exploited, for instance, by inducing high expression of TcprsTcprs that enhance the proliferation and activation of CAR-T cells. Conversely, the Tcprs that inhibit T cell proliferation and activation could be knocked down, B2M could be used to inhibit the allogeneic immune response, or CDK1 could be used to regulate tumor immune infiltration. In conclusion, it is possible to greatly enhance the antitumor activity of CAR-T cells and overcome their obstruction by the TME by targeted application of different top-ranked TCR genes.

#### 3.2.2 Inhibition of cancer progression

Fifteen Tcprs show positive correlations with cancer development, and eight are negatively correlated with cancer development. Two of these 21 genes show a tendency to either promote or inhibit development in different cancers. Two other Tcprs showed cancer-related characteristics (Table 3).

**Table 3:** Effects of TCR on cancer development.

Gene	Impact on cancer development	Correlation with cancer
CXCL12	+	/
GPN3	+	/
DCLRE1B	+	/
RAN	+	/
CYP27A1	+	/
IL1RN	+	/
HOMER1	+	/
B2M	+	/
CDK1	+	/
AKR1C4	+	/
ZNF830	+	/
CLIC1	+	/
NFYB	+	✓
AHNAK	+/−	/
FOSB	+/−	/
AHCY	–	/
GPD1	–	✓
ITM2A	–	/
CD19	–	/
CALML3	–	/
ADA	–	/

**Fig. 4:**
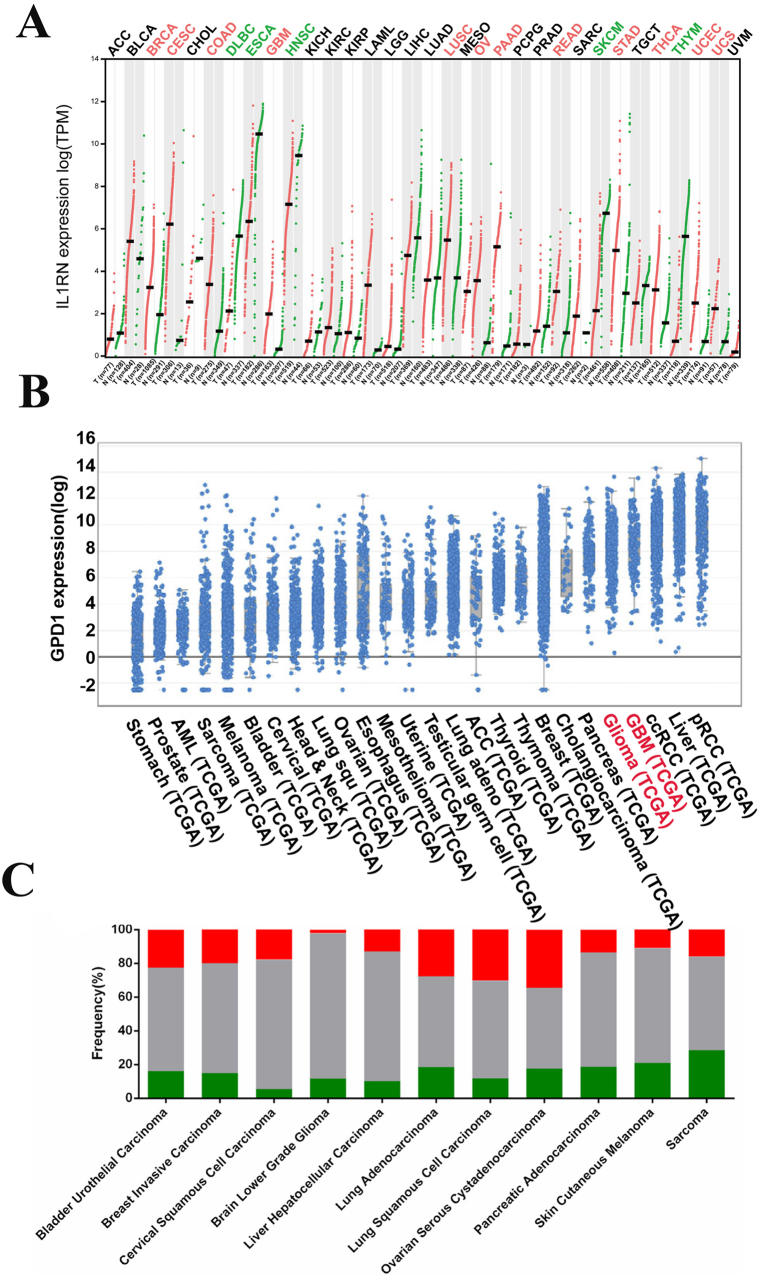
Differential expression of IL1RN and GPD1 in different cancers [95,118,119].

Many genes have been shown to contribute to different cancers. Chemokine CXCL12 and its receptor CXCR4 are associated with cancer development and poor prognosis [[77], [78], [79]]. Righi et al. observed that tumor cell growth was slower in vivo and tumor cell proliferation significantly decreased in vitro after the knockdown of CXCL12 [80]. Other studies have shown that CXCL12 can resist effector T cells and recruit suppressor cells at tumor sites [81], such as myeloid-derived suppressor cells [82]. Lara-Chacon et al. found that GPN3 has an essential role in the proliferation of cancer cells in a study of the relationship between GPN3 and breast cancer [83]. Cisplatin is a drug used to treat breast cancer. Lee et al. demonstrated that DCLRE1B knockdown could lead to breast cancer cell death by increasing susceptibility to cisplatin therapy [84]. Nuclear transcription factor Y subunit β (NFYB) is one of the three subunits of CCAAT and binds to transcription factor NF–Y, which is involved in the regulation of transcription of many cell cycle regulators, thereby affecting cell proliferation. Although cancer is the result of uncontrolled proliferation, it is possible to control cancer cell proliferation through NFYB. Studies have shown that knockdown of NFYB can induce cell growth in the G2 phase, thereby preventing cells from entering the M phase and ultimately affecting cell proliferation [85]. Feng et al. also demonstrated that NFYB could bind to the STK33 promoter to accelerate SYK33 transcription, promoting cisplatin resistance in DLBCL cells [86]. RAN is a member of the RAS oncogene family and encodes a small GTP-binding protein. RAN has been implicated in the development of many cancers because it regulates the rate at which molecules enter and leave the nuclear pore complex [87] and is also involved in the control of DNA synthesis and cell cycle progression. For example, RAN was found to be overexpressed in oral squamous cell carcinoma tissues, and knockdown of RAN induced apoptosis and cell cycle arrest, thereby delaying the proliferation of cancer cells [88]. RAN is overexpressed in many cancer cells [[89], [90], [91]] and is a potential anti-cancer therapeutic target. CYP27A1 encodes a member of the cytochrome P450 superfamily of enzymes. It is highly expressed in bone marrow cells and produces enzymes that synthesize 27HC, a major metabolite of cholesterol that promotes metastasis and progression of breast cancer [92]. Ma et al. found that breast cancer metastasis decreased after CYP27A1 knockout, which may indicate a promoting effect of CYP27A1 on cancer [93]. IL1RN encodes a member of the IL-1 cytokine family and is an endogenous natural antagonist of IL-1 [94] involved in the regulation of a variety of IL-1-related immune and inflammatory responses. IL1RN has been studied in several cancers. Xie et al. proved that IL1RN could promote the progression of thyroid cancer through immune-related pathways, and 12 cancer types were found to be significantly associated with increased expression of IL1RN in the pan-cancer relationship between IL1Rn and 33 cancers tested [95] (Fig. 4A). HOMER1 encodes a member of the Homeric family of dendritic proteins involved in the regulation of group 1 metabolic glutamate receptor function. HOMER1 protein has been observed in some cancer cells [96,97], and Cui et al. demonstrated that HOMER1 is highly expressed in colorectal cancer, possibly through the G3BP1 signaling pathway to promote cancer development [98]. B2M is a serum protein encoded by the housekeeping gene, which is associated with the major MHC class I heavy chain on the cell surface. The downregulation of the expression of MHC-I helps tumor cells to evade the surveillance of the immune system and improve their resistance to immunotherapy. Increased levels of B2M protein have been found in some tumors [99,100]. Wang et al. found that mesenchymal-stromal-cell-derived B2M promoted the occurrence and invasion of esophageal squamous cell carcinoma by enhancing epithelial–mesenchymal transition (EMT) [101]; Lhotakova et al. also found that the inactivation of B2M gene could reduce the proliferation, migration, and invasion of different types of tumor cells [102]. Many studies have shown that B2M is a prognostic factor for malignant tumors and can mediate tumorigenesis, angiogenesis, and metastasis [[103], [104], [105]]. B2M may be a powerful target for cancer therapy in the gene set of top Tcprs. CDK1 binds to cyclin and plays a part in cell cycle regulation, RNA transcription and processing, and other biological functions. Disruption of the CDK expression mechanism may lead to uncontrolled cell division and proliferation, so the role of CDKs in tumor formation is often reported [106]. Zhou et al. have demonstrated that the binding of CDK1 to fibroblast growth factor receptor 1 can affect the proliferation, invasion, and migration of breast cancer cells [107]. AKR1C4 encodes a member of the aldo/keto reductase superfamily, which in addition to participating in biochemical catalysis in cells has been shown to be involved in tumor development [108,109]. Studies have shown that AKR1C4 expression is increased in lung cancer and colorectal cancer [110,111] and associated with drug resistance through a carbonyl reductive metabolism inactivation [112]; it may also promote radioresistance in nasopharyngeal cancer [113]. ZNF830 encodes a nuclear zinc finger protein [114] that is involved in splicing precursor mRNA and is also involved in DNA damage repair mediated by homologous recombination. Chen et al. showed that ZNF830 could promote cell survival in response to DNA damage [115]. Therefore, cancer cells that overexpress ZNF830 may become resistant to chemotherapy or radiotherapy. CLIC1 is involved in regulating basic cellular processes. Studies have shown that CLIC1 is expressed in tumor cells and influences the proliferation and migration of cancer cells [116]; Ferician et al. have also shown that CLIC1 is expressed in endothelial cells lining tumor blood vessels, where it affects the stage and progression of tumors [117]. The above 13 types of Tcprs have been shown to promote different aspects of the development of cancer. Some genes, such as GPN3 and CYP27A1, have only been associated with one cancer type. These genes may require further pan-cancer analysis to demonstrate their value as cancer therapeutic targets. Genes expressed in multiple cancers, such as CXCL12, IL1RN, HOMER1, and B2M, could be used as target antigen sites or to modify CAR-T cells to improve CAR-T cell-specific recognition and reduce off-target effects.

In addition to the genes mentioned above, there are a number of Tcprs that are negatively associated with the development of cancer. For example, the T cell development marker ITM2A described above has a tumor suppressor role in breast and ovarian cancer and has been shown to negatively regulate cisplatin resistance in cervical cancer through the Notch signaling pathway [120]. AHCY encodes *S*-adenosine homocysteine hydrolase, which catalyzes reversible hydrolysis of *S*-adenosine homocysteine and plays an important part in determining the intracellular methylation level. Studies have demonstrated a relationship between AHCY and cancer [121], indicating that it could be used as a biomarker of cancer [122] or an anticancer therapeutic target [[123], [124], [125]]. It is important to note that AHCY may have completely different effects on different cancer types. For instance, AHCY is expected to inhibit the development of neuroblastoma [123] and breast cancer [121], induce apoptosis, and inhibit cell migration and adhesion in esophageal squamous cell carcinoma [126]. GPD1 encodes glycerol 3-phosphate dehydrogenase 1, which has a key role in carbohydrate and lipid metabolism. GPD1 has been identified as a tumor suppressor [118,127] that is activated in the early stage of tumor development and has an inhibitory role in cancers including breast cancer, lung cancer, prostate cancer, and glioblastoma [118,[119], [128], [129]] (Fig. 4B and C). A newly developed CD19CAR construct, which was previously mentioned, has shown promising results in the treatment of B-cell malignancies owing to high levels of CD19 in malignant and healthy B cells [130]. Hombach et al. therefore hoped to enhance the anti-tumor activity of CD19CAR-T cells in solid tumors using repeated co-stimulation of healthy CD19-expressing B cells, an approach that has been validated in ErbB2 cancer cells [131]. CALML3, which encodes calmodulin-like protein, has inhibitory effects in breast cancer and hepatocellular carcinoma [132,133]. ADA is associated with the cell surface glycoprotein CD26, and the combination of the two can reduce local levels of adenosine [134]. Adenosine is produced in large quantities in the TME, and downregulation of ADA enhances adenosine-mediated immunosuppression [135]. On the other hand, high ADA expression may inhibit tumor escape. All of the six Tcprs mentioned above have shown inhibitory effects on cancer development in various ways. The expression of cancer suppressors is significantly lower in cancer cells compared with that of genes that promote cancer development, which makes genetic testing difficult. As TCR pan-cancer research progresses, it should be possible to identify more genes that can inhibit the development of cancer. Accordingly, we make a bold suggestion: if CAR-T cells are armored with Tcprs capable of exerting tumor suppressive effects, could the specific tumor-killing effect be enhanced while benefiting the prognosis of patients? Further research is needed to determine the answer to this question.

In addition to the 19 genes described above that have positive or negative effects on cancer, two other genes showed both promoting and inhibiting effects. Gu et al. found that the proliferation, migration, and invasion of cancer cells were inhibited after the knockdown of AHNAK in bladder cancer (BCa) [136], but Cai et al. found that overexpression of AHNAK significantly inhibited the growth and metastasis of ovarian cancer cells [137]. The former can be explained by the mechanism by which AHNAK affects EMT as an RNA-binding protein. The latter may be related to the Wnt cascade, consistent with some studies showing that AHNAK can activate the TGFβ signaling pathway and thus function as a tumor suppressor [[138], [139], [140], [141]]. FOSB encodes a leucine zipper protein and, with members of the JUN family, forms transcription factor complex AP-1, which is involved in regulating cell differentiation, proliferation, and apoptosis [142]. Qi et al. found that FOSB was highly expressed in glioma, where it may promote development and migration [143], but Tang et al. proved that FOSB could inhibit the proliferation and migration of gastric cancer cells [144]. It is not impossible that these genes do play different parts in different cancer types, but more research is needed. These results also suggest that the relationship between Tcprs and cancer development will be clarified as research in this area progresses, including the identification of many genes that have not yet been found to be involved in cancer development.

#### 3.2.3 Improving persistence of CAR-T cells

**Fig. 5:**
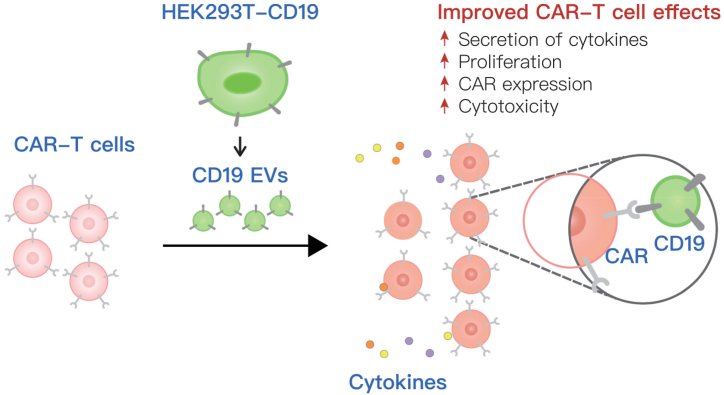
Schematic model of the effect of CD19 EV on CAR-T cell function [146].

The efficacy of CAR-T is related to the proliferation and persistence of CAR-T cells. However, of the 35 Tcprs, only six genes have been shown to be involved in enhancing CAR-T cell function. After identifying the top gene set of Tcprs, Legut et al. analyzed the top-ranked differential genes and found that CAR-T cells expressing LTBR showed better effects [16]. Seo et al. demonstrated that the survival and expansion of CAR-T cells derived from overexpression of BATF were greatly improved, while T cell exhaustion was inhibited and cytokine secretion was increased [145]. In addition, CD19 and ADA were found to enhance CAR-T cell persistence against T cell depletion [146,147] (Fig. 5). Other studies have shown that the destruction of B2M has a prominent effect in reducing the immune rejection of CAR-T cells, which can prevent the mismatch of HLAI molecules between donor and receptor [148].

## 4 Tcprs and cancer prognosis prediction

**Fig. 6:**
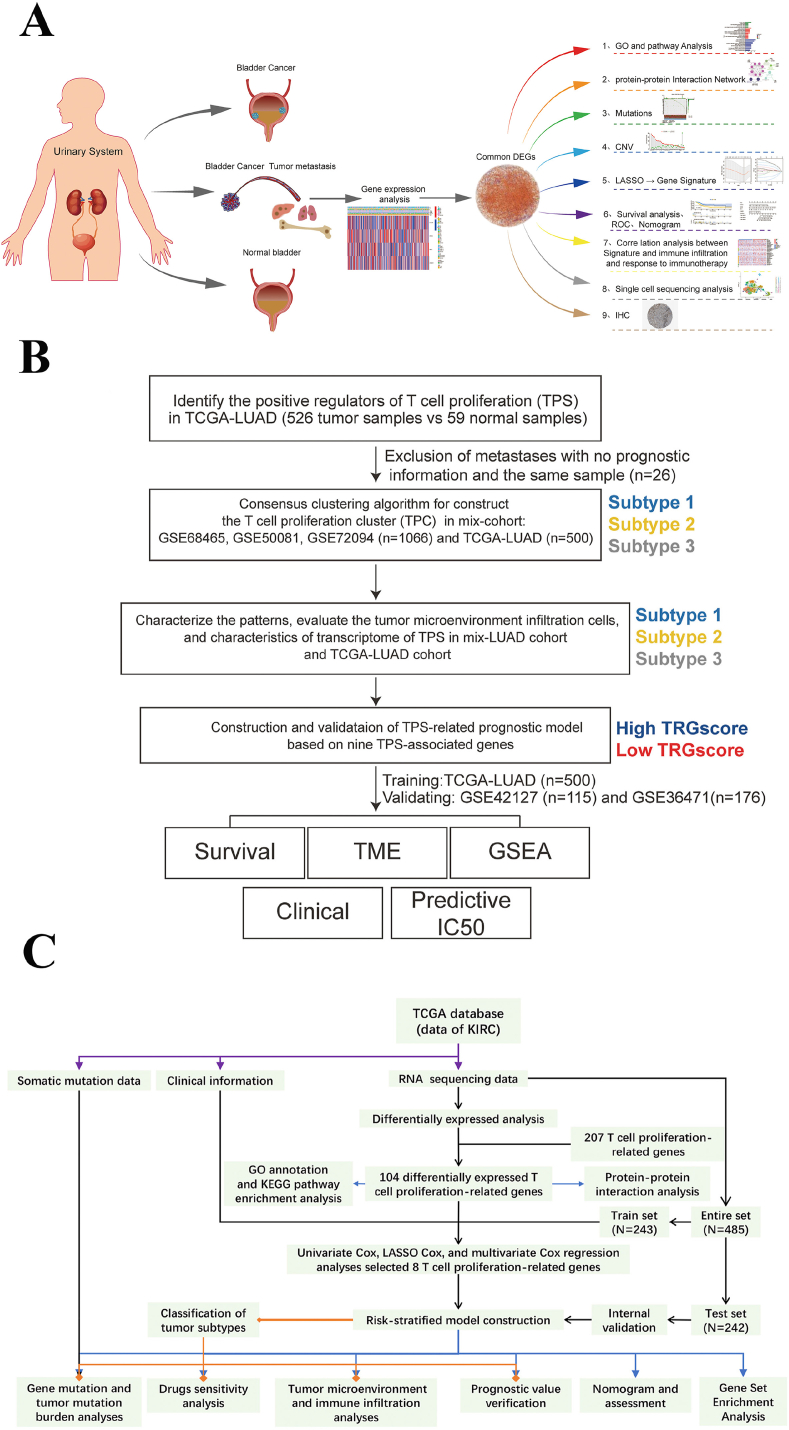
Flowcharts of three studies of Tcprs and cancer prognosis prediction [21,149,150].

The above mentioned effects of Tcprs on T cell function or CAR-T therapy may demonstrate the high relevance of Tcprs to cancer therapy. Some additional studies have been conducted in this area, all of which used Tcprs to predict the prognosis of patients with different cancers (Fig. 6). The research progress represented by these three papers is summarized below.

### 4.1 Bladder cancer

Hou et al. constructed a prognostic model of BCa based on the top TCR gene set first proposed by Legut et al. (Table 1) [16]. They finally screened out four genes, AHNAK, AHCY, HOMER1, and CXCL12, which they showed to be significantly correlated with the prognosis of BCa patients (Fig. 6A) [21].

Gene ontology and Kyoto Encyclopedia of Genes and Genomes enrichment analyses on the genes obtained from the difference analysis both showed that Tcprs were more concentrated in the important pathways of cell proliferation, leukocytes, B cells, lymphocyte differentiation, viral infection, and carcinogenesis. This suggests a role for Tcprs in T cell proliferation, activation, and cancer prognosis. They then used regression analysis to obtain a risk score prediction model involving four Tcprs. The results of the survival analysis showed the same trend in scores in the training and validation groups, with the high-risk patients having lower survival rates. In addition, to investigate the potential for clinical applications of the model, they developed a column graph to predict overall survival in patients with BCa over the next 1, 2, and 3 years. The results showed differences in the contributions of the four genes to the survival prediction score. The longest line segment was that of AHNAK, indicating that it contributes the most to the expected outcome of survival, whereas HOMER1 does the opposite.

After obtaining a reliable model, they further investigated the relationship between risk score and immune cell infiltration in the TME, the relationship between risk score and gene regulation, the relationship between risk score and tumor mutation burden (TMB), and the relationship between risk score and drug sensitivity. By single-sample gene set enrichment analysis (ssGSEA), they found that CXCL12 and AHNAK were associated with positive infiltration of most immune cells, whereas AHCY and HOMER1 were not. The ESTIMATE analysis showed that the immune score decreased with increasing risk score, whereas the stromal score showed the opposite trend. Overall, low-risk patients had higher expression of immune cells and a stronger immune response than high-risk patients. In addition, the risk score was also significantly correlated with TMB and drug therapy, and the TCR genes may have been associated with BCa resistance. These findings could provide potential targets for drug therapy of BCa.

After examining the predictive effect of the risk model on the prognosis of patients with BCa, they dug deeper into the mechanism of the influence of Tcprs on the TME of BCa based on evidence obtained from the above experiments. Single-cell sequencing analysis showed that CXCL12 was mainly enriched in fibroblasts of paraneoplastic tissues. Therefore, they concluded in their further study that it is CXCL12 that binds specifically to the CXCR4 receptor in T cells via paracrine secretion and promotes the proliferation of exhausted T cells in cancer tissue, thus promoting the occurrence and progression of bladder tumors.

In conclusion, this study established a new characteristic of Tcprs, revealed the correlation between Tcprs and the TME, and demonstrated that Tcprs may exert an impact on cancer development by controlling immune regulation. Moreover, the mechanism by which TCR-related gene CXCL12 promotes the development of BCa was discovered through single-cell analysis and provides new ideas for the clinical treatment of BCa.

### 4.2 Adenocarcinoma of lung

Li et al. published a paper identifying the role of a positive regulator of T cell proliferation (TPR) (Table 4) in the infiltration of lung adenocarcinoma (LUAD) cancer cells [149]. They collected TPR characteristics and screened nine TPRs (AGER, CYP27A1, CDK1, CADM1, FADD, ADA, LTBR, FYN, and CRTAM) to construct a risk model. Finally, they demonstrated the reliability of this risk model in the prognostic evaluation of LUAD patients (Fig. 6B).

**Table 4:** TPRs that overlap with the TCR set [149].

TPRs that overlap with the TCR set					
IFNL2	LTBR	IL1RN	CXCL12	IL12B	NFYB
BATF	FOSB	ATF6B	AHNAK	SLC10A7	CALML3
CLIC1	RAN	CDK2	MS4A3	CDK1	DBI
CYP27A1	AKR1C4	GPD1	GPN3	AHCY	ADA
ITM2A	HOMER1	DCLRE1B	MRPL51	LIG3	ZNF830

They constructed T cell proliferating clusters (TPCs) using unsupervised consensus clustering and divided the sample into three clusters, which were studied separately. Enrichment analysis showed that cluster 1 was significantly enriched in the carcinogenic pathway, cluster 2 was highly enriched in the carcinogenic and immune-activation-related pathways, and cluster 3 was enriched in the pathway negatively correlated with cluster 2. The low risk had more enrichment of immune functional pathways than the high risk group. Similarly, through regression analysis, they obtained a risk-score model involving nine TPRs. The survival analysis showed that patients with a high risk score had poorer survival. The column graph showed that TPR characteristics (risk score) and cancer stage contributed significantly to the survival prediction score.

Similar to the BCa-based study described earlier, they also investigated the relationships between risk scores and TME, gene regulation, TMB, and drug sensitivity. The three groups of TPCs showed different levels of immune infiltration by ssGSEA analysis (cluster 2 > cluster 1 > cluster 3). Cluster 2 had high immune scores and stromal scores and was referred to as the immune-inflammatory phenotype; cluster 1 was referred to as the intermediate phenotype; and cluster 3 was referred to as the immune desert phenotype. ESTIMATE analysis showed that four immune checkpoint genes (PDCD1, PDCD1LG2, CTLA4, and LAG3) associated with immune blockade were also differentially expressed in the three groups of TPCs (cluster 2 > cluster 1 > cluster 3). Patients in cluster 2 were therefore considered most likely to improve with immunotherapy. This was also demonstrated by TMB analysis and drug sensitivity analysis, as patients in cluster 2 or the low risk groups had higher TMB and were potentially more sensitive to immune checkpoint blockade therapies.

In summary, this study established characteristics of T cell proliferation and constructed three TPCs to characterize different LUAD immunophenotypes. The above results confirm the value of the TPR characteristic model in evaluating the prognosis of LUAD patients.

### 4.3 Clear cell renal cell carcinoma

Huang et al. established a prediction model for clear cell renal cell carcinoma (ccRCC) based on the fusion of 25 new genes discovered by Legut et al. and known T cell proliferation-related genes (TRGs) (Table 5) [150]. Eight TRGs (CTLA4, IL4I1, HHLA2, PRKCQ, IL20RB, HOMER1, DHPS, and TMEM131L) were selected and used to construct a prognostic model, which was shown to effectively predict prognosis, identify hot and cold tumors, and guide the treatment of ccRCC (Fig. 6C).

**Table 5:** Human TRGs that overlap with the TCR set [150].

Human TRGs that overlap with the TCR set					
ADA	AHCY	AHNAK	AKR1C4	ATF6B	BATF
CALML3	CDK1	CDK2	CLIC1	CXCL12	CYP27A1
DBI	DCLRE1B	FOSB	GPD1	GPN3	HLA-A
HOMER1	IFNL2	IL12B	IL1RN	ITM2A	LIG3
LTBR	MRPL51	MS4A3	NFYB	RAN	SLC10A7
ZNF830					

Enrichment analysis showed that TRGs were mainly enriched in immune-related and T cell proliferative activation pathways, indicating the potential applications of TRGs in the recognition of cold and hot tumors. Survival analysis further demonstrated that low-risk patients had good prognosis, and that age, pathological grade, tumor stage, and risk score were negatively correlated with good prognosis. The prediction results obtained using the column graph constructed based on the above characteristics were also consistent with real observations.

When studying the relationship between risk score and TME immune cell infiltration, the researchers found that the correlations of the immune score, microenvironment score, and cytotoxicity score in the high-risk group were higher than those in the low-risk group, possibly owing at least in part to the high level of immune cell infiltration in high-risk patients. In the drug sensitivity analysis, high-risk patients showed greater sensitivity to targeted agents, particularly sunitinib, whereas low-risk patients were sensitive to pazopanib and sorafenib. This suggests that particular drugs and immunotherapy techniques should be used for different types of patients. TMB analysis showed that none of the eight selected TRGs had significant mutations, although tumor suppressor gene VHL had the highest mutation frequency [151]. In addition, risk scores were positively correlated with TMB scores.

In summary, this study established a hierarchical model based on integrated modeling of old and new TRGs and discussed the value of TRGs for predicting prognostic outcomes in ccRCC. The results showed that the model was effective in guiding the treatment of ccRCC and laying a foundation for the discovery of TRGs as a new target for immunotherapy.

## 5 Conclusions and prospects

T cells are key to cancer therapy. However, immunotherapy for solid tumors is hampered by problems with target antigen selection, proliferation and persistence of CAR-T cells, and the TME and has not yet achieved good clinical results. In this review, we discuss the newly defined Tcprs, how they were discovered, and their role. T cells need three signals in order to be activated and exert their functions. Thus, we can use Tcprs to enhance T cell proliferation and activation. We also consider CAR-T therapy, which has shown remarkable results in cancer treatment. CAR-T technology can artificially specifically activate CD8^+^ T cells to specifically target and recognize tumor cells with the help of location-based navigation device CAR, but its applications in solid tumors have been hindered. There is evidence that many of the genes in which Tcprs are concentrated have an impact on T cell function and cancer development. Targeting these genes could improve the anti-tumor activity of CAR-T cells, enhance their persistence, and provide direction for target antigen selection. We have summarized strategies for enhancing CAR-T by targeting different Tcprs. In addition, we summarized research progress on the use of Tcprs in cancer prognosis prediction, a field in which there are great possibilities for the clinical application of Tcprs. It is important to note that some of the Tcprs have not been shown to promote the treatment of solid tumors, and that the use of CAR-T therapy to target Tcprs is a new idea that requires more theoretical research and clinical trials. In addition, there are differences among solid tumors, and specific mechanisms and treatment plans for applications of Tcprs need to be further explored. Enhancing CAR-T cells via the expression of different Tcprs may lead to the development of a new generation of cell therapies, but more strategies are needed to improve the safety and efficacy of this approach.

## Funding

This research was funded by the 10.13039/501100001809National Natural Science Foundation of China (No. 62371318, No. 62001090, No. 62250028, No. 62131004) the Sichuan Provincial Science Fund for Distinguished Young Scholars (No. 2021JDJQ0025), the Municipal Government of Quzhou (No. 2022D040), and Fundamental Research Funds for the Central Universities of 10.13039/501100004912Sichuan University (No. YJ2021104).

## Data availability statement

Data included in article/supplementary material/referenced in article.

## Additional information

No additional information is available for this paper.

## CRediT authorship contribution statement

**Jiayu Li:** Writing – review & editing, Formal analysis, Data curation. **Shuhan Ma:** Writing – review & editing. **Hongdi Pei:** Writing – review & editing. **Jici Jiang:** Writing – review & editing. **Quan Zou:** Writing – review & editing, Funding acquisition. **Zhibin Lv:** Writing – review & editing, Funding acquisition, Formal analysis, Data curation, Conceptualization.

## Declaration of competing interest

The authors declare that they have no known competing financial interests or personal relationships that could have appeared to influence the work reported in this paper.
